# *Campylobacter*-associated hospitalisations in an Australian provincial setting

**DOI:** 10.1186/s12879-020-05694-0

**Published:** 2021-01-06

**Authors:** Cameron R. M. Moffatt, Karina J. Kennedy, Linda Selvey, Martyn D. Kirk

**Affiliations:** 1 National Centre for Epidemiology and Population Health, Research School of Population Health, Australian National University, Canberra, ACT 2602 Australia; 2 Department of Microbiology, Canberra Hospital and Health Services, Canberra, ACT Australia; 3 School of Public Health, University of Queensland, Brisbane, QLD Australia

**Keywords:** *Campylobacter*, Infectious gastroenteritis, Hospitalisation, Length of stay, Comorbidity, Epidemiology, Clinical coding, Data accuracy

## Abstract

**Background:**

*Campylobacter* spp. infections are a globally important cause of enterocolitis, causing substantial morbidity. Capturing accurate information on hospitalisations is challenging and limited population-level data exist to describe the clinico-epidemiological characteristics of hospitalised cases.

**Methods:**

Hospital administrative and laboratory datasets were linked to identify *Campylobacter*-associated hospitalisations between 2004 and 2013. Accuracy of morbidity coding was assessed using laboratory diagnosis as a gold standard, with health department surveillance data used to calculate population-based rates. Additional patient-level data were collected via review of medical records. Descriptive statistics were used to assess changes in rates and proportions and to assess relationships between key variables including age, length of stay, comorbidity and complications.

**Results:**

In total 685 *Campylobacter*-associated hospital admissions were identified, with the sensitivity of morbidity coding 52.8% (95% CI 48.9–56.7%). The mean annual rate of hospitalisation was 13.6%. Hospitalisation rates were higher for females across most age-groups, while for both genders marked increases were observed for those aged ≥60 years. Median admission age was 39.5 years, with an average length of stay of 3.5 days. Comorbidities were present in 34.5% (237/685) of admissions, with these patients more likely to develop electrolyte disturbances, hypotension, renal impairment or acute confusion (all *p* < 0.001). Bacteraemia and acute kidney injury were observed in 4.1% (28/685) and 3.6% (23/685) of admissions, respectively. Inpatient mortality was low (0.15%).

**Conclusion:**

Under reporting of *Campylobacter*-associated hospitalisations is substantial but can be improved through data linkage. We observed demographic differences among those hospitalised but further work is needed to determine risk factors and predictors for hospitalisation.

## Background

In high-income settings, *Campylobacter* spp. are the most frequently reported bacterial enteric pathogen, with increasing disease incidence observed in the United States (US), European Union and Australia [1–3]. Campylobacteriosis typically presents as an acute enterocolitis, characterized by profuse diarrhoea and abdominal pain, along with nonspecific symptoms including fever, myalgia and lethargy [4]. While uncommon, complications including bacteraemia and post-infectious sequelae such as reactive arthritis and Guillain-Barré syndrome also occur [5–7].

National (and provincial-level) enteric surveillance systems are frequently passive, capturing only limited data on outcomes such as hospitalisation and death [8]. Consequently, estimating hospitalisation has become an established method for describing foodborne-related disease burden [8–10]. Nevertheless, differences in data sources and the assumptions underpinning these methods make intercountry comparison of hospitalisation rates challenging [8].

Given increasing disease incidence and concerns regarding accuracy of estimates, it is important to re-examine *Campylobacter*, with the aim of this study to quantify associated hospitalisations and describe their epidemiological and clinical characteristics, including the spectrum of illness, risk factors and associated outcomes.

## Methods

### Setting

The Australian Capital Territory (ACT) is a self-governing territory in south-eastern Australia, surrounded by the state of New South Wales (NSW). It contains the national capital Canberra, with an estimated population of 410,000 [11]. Two public hospitals service the ACT. Canberra Hospital, an acute care teaching facility of approximately 600 beds, acts as the tertiary referral centre to both the ACT and surrounding parts of NSW, while Calvary Public Hospital, is a smaller acute care teaching facility of approximately 250 beds. Both hospitals support around 550,000 people in the ACT and surrounding NSW. Three private hospitals operate within the ACT but only the two public hospitals have emergency departments (EDs).

### Outcome definition and objectives

A *Campylobacter*-associated admission was defined as any inpatient episode clinically and temporally linked to a hospital-derived *Campylobacter* isolate. Our study objectives included examining admission counts and calculating hospitalisation rates, describing the clinical and demographic characteristics of admissions and assessing the impact of admission characteristics, including age, gender and comorbidity, on outcomes including length of stay (LOS) and time to admission.

### Data sources and data collection

#### Hospital administrative data

We obtained public hospital admissions data between 01 January 2004 and 31 December 2013, where the International Classification of Diseases (ICD) morbidity code ‘A045 *Campylobacter* enteritis’ was recorded as a primary or secondary diagnosis. Included were unit record (UR) and admission numbers, birth dates, gender, admission and discharge dates, admission sources, admission units and discharge destinations. LOS was calculated by subtracting admission from discharge dates, with reporting in whole days. Same day admissions and discharges received a LOS of 1 day. Private hospital admissions were not sought due to the acute nature of campylobacteriosis and absence of private hospital EDs.

#### Hospital microbiology data

Hospital microbiology data for the period 01 January 2004 to 31 December 2013 were used to identify admissions where *Campylobacter* spp. were isolated from clinical samples but where ICD code ‘A045’ was not assigned. UR and admission numbers, birth dates, gender, isolation dates, specimen type (e.g. faeces, blood), and hospital, ward and unit identifiers were provided, facilitating linkage with administrative data. All *Campylobacter* diagnoses were made via culture, with routine speciation not performed before 2013. Details on concurrent isolation or detection of additional enteric pathogens were also provided.

#### Medical record data

Medical records were reviewed to confirm admissions and retrieve details unavailable via administrative data sets. This included the presence or absence of signs and symptoms, onset dates (defined as the date of earliest diarrhoea), time to admission (defined as difference in whole days between the onset and admission date), travel history, comorbidities, complications and discharge summaries. Time to admission was calculated for initial acute admissions and not for readmissions or inter-state hospital transfers.

Key signs of infection sought included tachycardia (resting heartrate ≥110 bpm), hypotension (systolic blood pressure < 90 mmHg), electrolyte imbalance (levels outside of typical reference range for serum sodium of 135–145 mmol/L and/or potassium of 3.5–5.2 mmol/L) and acute renal impairment (levels above typical reference range for serum creatinine in males of 60–110 μmol/L and females of 45–90 μmol/L and/or urea of 3.0–8.0 μmol/L). Charlson Co-morbidity Index (CCI) scores [12] were calculated as a marker of comorbidity.

#### ACT surveillance notifications and emergency presentation data

*Campylobacter* notification data between 01 January 2004 and 31 December 2013 were obtained from the ACT Government Health Directorate. After identifying ACT resident *Campylobacter*-associated admissions, we sought to link these back to notification data using birth date, gender, postcode and specimen collection date. This enabled calculation of a hospitalisation rate for ACT campylobacteriosis cases. Hospital-generated microbiology data also enabled matching of non-admitted ED presentations to notification data to calculate an ED presentation rate for ACT residents.

### Exclusions

Admissions were excluded if additional enteric pathogens were detected or isolated from a sample, if diarrhoeal symptoms commenced ≥48 h after admission and if *Campylobacter* spp. were isolated during a planned or elective admission. The exception to this was for planned admissions involving bone marrow or stem cell transplantation. Given the importance of microbial translocation and overgrowth in patients being treated for haematological malignancies [13], we postulated this patient group had underlying *Campylobacter* spp. carriage at the time of admission.

### Analysis

Hospitalisation and non-admitted ED presentation rates were calculated using ACT resident admission and surveillance data. Sensitivity, specificity, positive and negative predictive values for ICD coding using laboratory diagnosis as the gold standard were calculated. Age and gender specific rates of hospitalisation were calculated for ACT residents. Changes in rates and proportions over time were assessed using linear tests for trend, while two-sample tests of proportions were used to assess equality between groups. Relationships between outcome and independent variables were assessed using Pearson’s chi-squared or Fisher’s exact tests. Equality of median tests and Spearman’s correlations were used to analyse non-normally distributed continuous data including age, LOS, CCI score and time to admission.

## Results

### Linking hospital administrative data to laboratory data

**Fig. 1:**
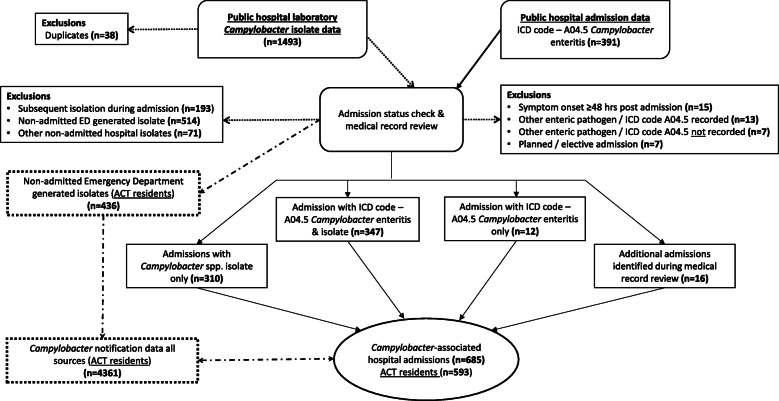
Flow diagram detailing selection of 685 *Campylobacter*-associated admissions and emergency department presentations via linkage of public hospital administrative data and laboratory data

We identified 685 *Campylobacter*-associated admissions during the study period **(**Fig. 1**)**. Hospital administrative data identified 52% (359/685) of admissions, laboratory data 45% (310/685) with the remaining 16 admissions identified via medical record review. For admissions identified via administrative data, 84% (300/359) had *Campylobacter* enteritis recorded as a primary diagnosis code. For admissions identified via laboratory data, primary diagnosis codes were available for 81% (250/310), with two-thirds (168/250) assigned a non-specific gastroenteritis code. Sensitivity of morbidity coding using laboratory diagnosis as the standard was 52.8% (95% CI 48.9–56.7%), and specificity was 57.1% (95% CI 37.2–75.5%). The positive and negative predictive values were 96.7% (95% CI 94.2–98.3%) and 4.9% (95% CI 2.8–7.8%).

**Fig. 2:**
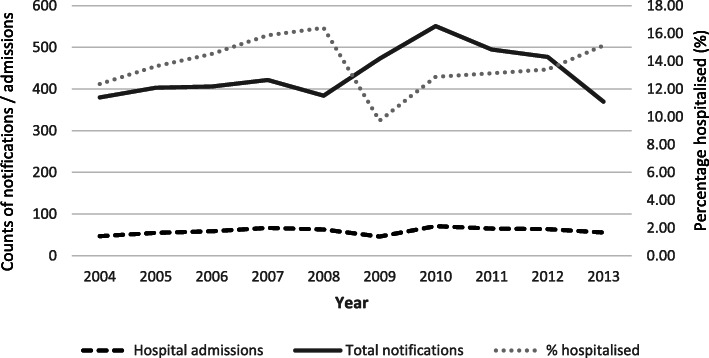
Counts of *Campylobacter* notifications and associated public hospital admissions among Australian Capital Territory residents, 2004 to 2013

ACT residents comprised 86% (593/685) of admissions, with a hospitalisation rate of 13.6% (593/4361) (Fig. 2). EDs were the admission source for 94% (555/593) of ACT resident admissions, with a further 10% (436/4361) of ACT *Campylobacter* notifications linked to at least one non-admitted ED presentation. No trends over time were observed in the proportions of *Campylobacter*-associated admissions or non-admitted ED presentations. Admissions did not appear to show any obvious seasonal pattern (data not shown).

### Descriptive characteristics

#### Age and sex characteristics

**Fig. 3:**
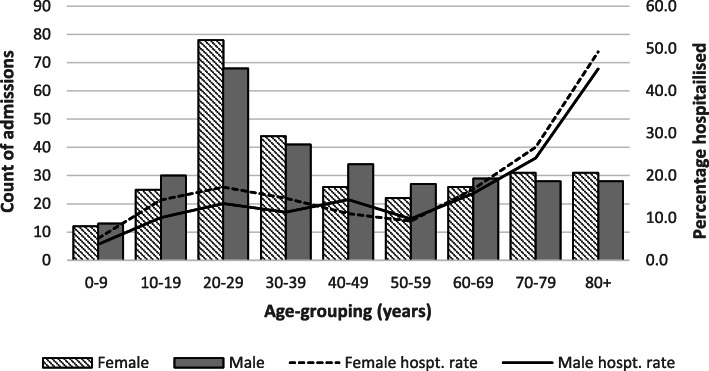
*Campylobacter*-associated public hospital admissions and percentage hospitalised by age-group and gender among Australian Capital Territory residents, 2004–2013

Age and sex characteristics are shown in Table 1. Males were older (*M* = 41.2 years, range < 1 to 92.3) than females (*M* = 38.5, range < 1.0 to 92.3) but this difference was not significant. For ACT resident admissions, the median age was 38.4 years (range < 1.0 to 92.3 years), compared with 31 years (range < 1.0 year to 99.0 years) among ACT community cases (Χ^2^ = 17.7, *p* < 0.001). Non-ACT residents were significantly older (*M* = 50.8, range < 1 year, maximum 90.8 years, Χ^2^ = 5.5, *p* = 0.02). Hospitalisation rates increased noticeably among patients aged ≥60 years. The proportion of females admitted across most age-groups was higher than for males, although these differences were small and non-significant (Fig. 3**).**

**Table 1:** Demographic and clinical characteristics of 685 *Campylobacter*-associated admissions in Australian Capital Territory (ACT) public hospitals, 2004–2013

Gender	
Male (n, %)	350 (51.0)
Female	335 (49.0)
ACT resident (%)	593 (86.6)
Median (*M*) age in years for all admissions (range)	39.7 (< 1.0–92.3)
Indigenous Australian (%)	7 (1.0)
Age-groups % (hospitalisation rate per 100,000 population^a^)	
0–9 years (*n* = 30)	4.38 (*5.67*)
10–19 years (*n* = 67)	9.78 (*11.91*)
20–29 years (*n* = 158)	23.07 (*23.72)*
30–39 years (*n* = 93)	13.58 (*15.58*)
40–49 years (n = 67)	9.78 (*11.91*)
50–59 years (*n* = 57)	8.32 (*11.17*)
60–69 years (*n* = 68)	9.93 (*19.53*)
70–79 years (*n* = 74)	10.80 (*39.40*)
80+ years (*n* = 71)	10.36 (*65.35*)
Acute care admission (%)	673 (98.3)
Average length of stay (range)	3.5 days (1.0–38.0)
Same day acute admissions (*n* = 268)	1.0 day
Non-same day acute admissions (*n* = 405)	4.9 days (2.0–38.0)
Other separation types (*n* = 12)	8.6 days (1.0–23.0)
Median (*M*) length of stay (range)	2.0 days (1.0–38.0)
Non-same day acute admissions (*n* = 405)	4.0 days (2.0–38.0)
Comorbidity as per Charlson Comorbidity Index (CCI) (count, (percentage))	237 (34.5)
CCI score (n, %)	
1	86 (36.3)
2	81 (34.2)
> 2 (%, range)	70 (29.5, 3–12)
Median (*M*) time to admission from illness onset for acute admissions ^*b*^ (range)	3.0 days (< 1.0–23.0)
Key signs of infection ^c^	
Electrolyte disturbance (n, %)	410 (60.2)
Tachycardia	241 (35.4)
Renal impairment	127 (18.7)
Hypotension	87 (12.8)
History of recent overseas travel (n, %)	11 (1.6)
Re-admission ≤28 days related to campylobacteriosis, including non-acute status changes (n, %)	38 (5.6)
Intensive care unit admission (n, %)	10 (1.5)
Surgical or invasive diagnostic procedure related to campylobacteriosis (n, %)	45 (6.6)
Death during admission or ≤ 28 days post discharge (n, %)	5 (0.7)
Blood sample taken for culture (percentage *Campylobacter* positive)	333 (7.5)
Antimicrobial therapy during admission (n, %)	219 (32.0)
^a^ACT residents only ^b^636 observations ^c^681 observations

### Prior care, source of admission and admitting unit

Twenty-seven percent (187/684) of admissions consulted a general practitioner (GP) prior to hospitalisation, while 13.4% (92/685) were hospitalised after an earlier non-admitted ED presentation. There were 29 admissions (4.2%) preceded by both GP consultations and non-admitted ED presentations. EDs were the admission source for 91.8% (629/685) of admissions, with the remainder via community or outpatient admissions, inter-hospital transfers or care type changes.

#### Length of stay (LOS)

The median LOS for *Campylobacter*-associated admissions was 2.0 days (range 1.0–38.0), with no gender or residency differences observed. Exclusion of same day Emergency Medicine unit admissions (*n* = 180), markedly increased the median LOS (*M* = 3.0 days, range 1.0–38.0) and average LOS (Table 1). A moderately positive correlation between age and LOS was observed (Spearman’s = 0.3557, *p* = < 0.001). Higher median LOS were observed for units including Oncology, Haematology, and Geriatrics, while significant correlations between increasing age and LOS, were observed among General Medicine, Renal Medicine and Gastroenterology admissions (data not shown).

### Comorbidities

Comorbidities were documented in 34.6% (237/685) of admissions. The most prevalent were diabetes 30.8% (73/237), malignancies (including solid and disseminated tumours, blood and lymphatic cancers) 27.4% (65/237), chronic kidney disease 19.0% (45/237), cerebrovascular disease 17.3% (41/237) and history of myocardial infarction 13.1% (31/237). The median CCI score was 2 (range 1 to 12). Males comprised 53.6% (127/237) of admissions with comorbidities but no gender differences in CCI score were observed. Admissions with comorbidities were older, having a median age of 66.7 years (range 3.5 to 92.3 years) versus 28.8 years (range < 1.0 to 92.3) (Χ^2^ 202.0, *p* < 0.001). A positive correlation between increasing age and CCI scores was observed (r_s_ = 0.18, *n* = 237, *p* < 0.001). LOS were longer in admissions with comorbidities, with a median of 4.0 days (range 1.00 to 38.0 days) versus 1.0 day (range 1.0 to 22.0, Χ^2^ 68.93, *p* < 0.001). A positive correlation between increasing LOS and CCI scores was demonstrated (r_s_ = 0.25, *n* = 237, *p* < 0.001), while a larger proportion of admissions with comorbidities had times to admission greater than the 3 day median (Χ^2^ 4.55, *p* = 0.03).

### Time to admission

The median time between onset and hospitalisation for initial acute admissions (*n* = 662) was 3.0 days (range < 1.0 to 24.0). A higher proportion of females had times to admission greater than the median compared to males (Χ^2^ 6.03, *p* = 0.01). A positive correlation between increasing age and time to admission was observed (r_s_ = 0.15, *n* = 662, *p* < 0.001).

### Key signs of infection

These were observed in 74.5% (510/685) of admissions (Table 1**)**, with electrolyte imbalance being the most commonly reported. Renal impairment was associated with any CCI-linked comorbidity (Χ^2^ = 128.30, *p* < 0.001) along with male gender (Χ^2^ = 12.56, *p* < 0.001). Hypotension (Χ^2^ = 27.91, *p* < 0.001) and electrolyte imbalances (Χ^2^ = 22.24, *p* < 0.001) were also associated with underlying comorbidities. Correlations between the presence of multiple signs of infection (including electrolyte imbalance, tachycardia, renal impairment and hypotension), and increasing age (r_s_ = 0.32, *n* = 682, *p* < 0.001) and LOS (r_s_ = 0.22, *n* = 682, *p* < 0.001) were observed. Admissions without comorbidities were more likely to have bloody diarrhoea, self-reported fevers, abdominal pain and headaches documented, while key signs of infection and *Campylobacter*-associated readmissions occurred more frequently in admissions with comorbidities (Table 2).

**Table 2:** Comparison of signs, symptoms, and other outcomes among *Campylobacter*-associated admissions with and without documented comorbidities (as per Charlson Co-morbidity Index*)*

	Comorbidity present (***n*** = 237)						
Signs and symptoms associated with enterocolitis	Yes	%	No	%	Total	Χ^**2**^	***p***-value
Diarrhoea	231	98.7	444	99.1	675	0.23	0.63
Bloody diarrhoea	28	18.7	121	35.2	149	13.51	< 0.001
Self-reported fever	129	58.1	341	78.0	470	28.57	< 0.001
Fever (≥38 °C)	114	50.0	224	51.4	338	0.11	0.74
Abdominal pain	161	81.7	403	95.7	564	33.00	< 0.001
Nausea	142	79.8	311	87.6	453	5.70	0.02
Vomiting	114	58.8	234	56.5	348	0.27	0.60
Myalgia / arthralgia	35	76.1	122	87.1	157	3.22	0.07
Malaise	102	91.9	152	96.8	254	3.18	0.07
Headache	53	66.3	149	89.8	202	20.31	< 0.001
**Key signs and symptoms of infection**							
Hypotension	52	22.3	35	7.8	87	27.94	< 0.001
Tachycardia	89	38.2	152	33.9	241	1.22	0.27
Electrolyte imbalance	169	72.5	241	53.8	410	22.46	< 0.001
Renal impairment	98	42.1	29	6.5	127	127.95	< 0.001
Acute confusion	34	14.6	4	0.9	38	54.60	< 0.001
Bacteraemia	17	7.2	11	2.5	28	8.80	< 0.01
**Other outcomes**							
ICU admission	10	4.2	0	–	10		< 0.001^a^
Death within 28 days of discharge	5	2.1	0	–	5		< 0.01^a^
*Campylobacter*-associated readmission	22	9.3	16	3.6	38	9.65	0.002

^a^ Fisher’s exact test

### Complications and other outcomes

A diversity of complications were documented at discharge, with systemic disease and acute kidney injury the most frequently recorded (Table 3). The median age for admissions with systemic disease was 60.8 years (range 12.0 to 90.1) compared to 38.6 years (range < 1.0 to 92.3) among other *Campylobacter*-associated admissions (Χ^2^ 9.60, *p* = 0.002). The median LOS was 3.5 days (range 1.0 to 28.0), being significantly greater than other admissions (*M* 2.0 days, range 1.0 to 38.0, Χ^2^ = 7.70, *p* < 0.01). An association between comorbidity per se and systemic illness was observed (Χ^2^ 8.80, *p* < 0.01) with individual associations seen for admissions with liver disease (Χ^2^ = 50.78, *p* < 0.001) and leukaemia (Χ^2^ = 5.67 *p* = 0.02).

**Table 3:** Complications recorded at discharge among *Campylobacter*-associated hospital admissions in the Australian Capital Territory, 2004–2013

	Frequency (%)
**Extra-intestinal / systemic disease**	
Bacteraemia (laboratory proven)	28 (4.1%)
Sepsis (discharge diagnosis)	19 (2.8%)
**Intestinal complications**	
Appendicitis (histologically confirmed)	8 (1.2%)
Mesenteric adenitis	6 (0.9%)
Acute pancreatitis	4 (0.6%)
Acute cholecystitis	1 (0.2%)
**Other significant complication**	
Acute kidney injury	23 (3.4%)
Acute myocardial infarction	6 (0.9%)
Delirium	3 (0.4%)
Metabolic acidosis	3 (0.4%)
Atrial fibrillation	2 (0.3%)
Rhabdomyolysis	2 (0.3%)
Febrile seizure	1 (0.2%)
Hypovolemic shock	1 (0.2%)
Spontaneous abortion	1 (0.2%)
Perforated diverticulum	1 (0.2%)
Peritonitis	1 (0.2%)
Pulmonary embolism	1 (0.2%)
Thrombocytopaenia	1 (0.2%)
Transient Ischaemic Attack	1 (0.2%)
**Reactive complication**	
Guillan-Barré Syndrome	2 (0.3%)
Myopericarditis	2 (0.3%)
Reactive arthritis	1 (0.2%)
IgA nephropathy	1 (0.2%)
**Unnecessary surgery**	
Appendicectomy (normal histology)	8 (1.2%)

Acute Kidney Injury (AKI) was diagnosed among 3.4% (23/685) of admissions. These were significantly older (*M* 73.9 years, range 33.2 to 87.5, median test Χ^2^ = 19.92, *p* < 0.001), and took longer to present following symptom onset (*M* 5.0 days, range < 1.0 to 14, Χ^2^ = 16.87, *p* < 0.001). No differences in gender, LOS or CCI score were observed among acute admissions without AKI. Renal impairment was observed in 18.7% (123/672) of acute admissions. This group was also older (*M* 73.8 years, range 23.7 to 92.3, Χ^2^ = 101.51, *p* < 0.001), had longer LOS (*M* 4.0 days, range 1.0 to 31.0, Χ^2^ = 27.91, *p* < 0.001), and a greater proportion of admissions with CCI scores > 2 (*M* 2, range 1 to 12, Χ^2^ = 29.57, p < 0.001). A significant association with male gender was observed (Χ^2^ = 12.43, *p* < 0.001).

Readmissions ≤28 days after discharge occurred among 9.6% (66/685) of admissions, with 57.6% (38/66) being *Campylobacter*-associated. These included 27 admissions for ongoing enterocolitis and 11 for non-acute care. The median age and LOS for acute readmissions were 36.9 years (range 10.1 to 85.6) and 1 day (range 1 to 7 days). No gender-based associations were observed.

Surgical or invasive diagnostic procedures (*n* = 52) were performed during 6.6% (45/685) of admissions. Colonoscopies accounted for 46% (24/52), appendectomies 31% (16/52) and gastroscopies 19% (10/52) of procedures. The median age of those undergoing procedures was 28.8 years (range 12.0 to 81.4), significantly lower than for other acute admissions (*M* = 40.1 years, range < 1.0 to 92.3, Χ^2^ = 8.54, *p* < 0.01), while median LOS was 4 days (range 1 to 22), being compared to 2 days (range 1 to 38 days, Χ^2^ = 20.70, *p* < 0.001).

## Discussion

In this study, only one out of every two *Campylobacter* infections was identified via hospital discharge data. While hospitalisation is considered a marker of severity for foodborne bacterial infections [14], estimating enteric pathogen hospitalisations is challenging given concerns regarding the completeness of surveillance data and the accuracy of pathogen-specific diagnoses and morbidity coding [15]. By identifying all hospital-derived *Campylobacter* isolates and linking these to inpatient admissions and surveillance data we calculated the sensitivity of morbidity coding associated with *Campylobacter* infection to be 52%, indicating substantial measurement error. This has been demonstrated in similar settings with US [15] and Swedish [16] studies reporting appropriate use of ICD coding among hospitalised campylobacteriosis cases to be 43 and 51% respectively.

The 14% hospitalisation rate we reported is high, reflecting *Campylobacter*-associated morbidity in Australia [3, 9]. Intercountry comparisons of hospitalisations however reflect differences in incidence and data capture. In New Zealand, 12.6% of cases were reported as hospitalised (although data was complete for only 62.2% of cases) [17] while in Germany, where hospitalisation status is routinely collated, the rate was 10% [18]. Elsewhere hospitalisation rates have ranged from estimates of 5.1% in Canada [19] to 26.9% in a Swedish study [16].

Population characteristics of campylobacteriosis in developed settings are typified by increased disease incidence in males and a bimodal age-distribution, with peaks in children aged < 5 years and adults < 45 years [20]. We observed higher female admission rates across all age groupings, a finding reported in a number of studies examining hospitalisation for enteric infections [21, 22]. Reasons for this remain uncertain but inadequate social support for older women has been proposed as a contributing factor [21], while our study suggests females may delay seeking care. The increase in admissions aged 20–29 years reflects higher case numbers, with *Campylobacter*-associated hospitalisation rates increasing most dramatically after 60 years. These observations are consistent with reported changes in the population structure for campylobacteriosis in Australia and similar settings [3, 23], highlighting the potential hospitalisation costs for the elderly and necessitating a need for age-specific interventions [24].

Signs and symptoms observed were typical of campylobacteriosis, with most admissions having diarrhoea and abdominal pain recorded [4]. Reports suggest older campylobacteriosis cases may report some signs and symptoms, including bloody diarrhoea, abdominal pain, vomiting and fevers less frequently [25]. Our data support this juxtaposition, although it seems unlikely that age-related differences in presentation would impact a *Campylobacter* diagnosis due to the proclivity of stool testing.

Reported LOS vary dependent upon the measures of central tendency used. We reported mean and median LOS of 3.5 and 2.0 days, while for acute non-same day admissions the mean and median LOS were 4.9 and 4.0 days. These appear comparable to findings elsewhere [19, 26]. Similarly we observed cases with bacteraemia or underlying comorbidities to have longer LOS [27, 28]. Notably 40% of admissions involved same day care, illustrating the spectrum of hospitalisation, while linkage of surveillance and laboratory data revealed 10% of non-hospitalised cases presented to EDs for non-admitted acute care. Reasons for this ED burden might include perceived symptom severity and lack of access to a GP [29].

One third of patients had a comorbidity, a proportion similar to studies describing hospitalisations for infectious gastroenteritis [27]. These patients were generally older, took longer to present and had longer LOS. Diabetes was the most frequently recorded comorbidity, followed by malignancies and chronic kidney disease. While some evidence suggest diabetes and chronic kidney disease increase risk for *Campylobacter* enterocolitis [30, 31], relationships between *Campylobacter* infection and malignancies are more well established [32, 33]. Admissions with comorbidities experienced electrolyte imbalances, renal impairment, hypotension, acute confusion and acute readmission more frequently.

Half of acute admissions had blood cultures, with a positivity rate of 7.5%. These admissions involved older patients with higher comorbidity levels, although associated mortality was low, features consistent with findings elsewhere [28, 34]. Although no deaths occurred among blood culture positive cases, there was one death in a case diagnosed with blood culture negative sepsis.

Our study suggests renal impairment may occur more frequently in patients’ hospitalised with campylobacteriosis. This is supported by studies describing increased renal dysfunction among hospitalised gastroenteritis cases [30, 35, 36]. Furthermore comorbidities including kidney disease, hypertension, diabetes and a history of organ transplantation have been shown to increase AKI risk among patients with infectious diarrhoea [30].

Around 7% of admissions underwent surgical or invasive diagnostic procedures, with appendectomies and colonoscopies most frequently performed. Among appendix specimens, 50% showed histological evidence of inflammation, although specimens were not microbiologically tested. The issue of *Campylobacter*-associated appendicitis remains contentious, with a study re-examining archived appendices using nucleic acid testing detecting *C. jejuni* in 22% of specimens [37].

Limitations with our study include the cross-sectional design and generalisability of findings given the small provincial source population [38]. Local epidemiology is also an important consideration, with ACT campylobacteriosis rates higher than most other Australian states [3]. Differences in health care utilisation also require consideration [39]. We assumed hospitalisation would most likely occur in the public sector, with the extent of private hospital admissions being uncertain. Given the size and role of private hospitals in Australia’s health care system [40], our calculated rate of hospitalisation for ACT residents may be an underestimation. We were unable to estimate predictors of hospitalisation, however access to integrated Australian hospital and primary care data is lacking [41], making comparisons challenging. While plausible, our findings regarding AKI and renal impairment require further assessment. AKI diagnoses were not validated against a standard, e.g. Kidney Disease: Improving Global Outcomes (KDIGO) criteria [42], while our assessment of renal impairment via serum creatinine levels was simplified.

## Conclusion

Hospitalisation data provides an important indicator of the burden and severity of campylobacteriosis. Improving the accuracy and completeness of *Campylobacter*-associated hospitalisation data, in conjunction with detailed clinico-epidemiological characterisation is of tangible benefit to clinicians and public health decision makers. Undertaking observational studies, including genomic assessment of virulence factors, would improve understanding of patient and pathogen specific predictors of hospitalisation.

## Data Availability

The data that support the findings of this study are available from the ACT Government Health Directorate and Calvary Health Care (Bruce). Restrictions apply to the availability of these data, which were used under approvals for the current study and so are not publicly available. Data are however available from the authors upon reasonable request and with permission of the ACT Government Health Directorate and Calvary Health Care (Bruce).

## Abbreviations

ACT: Australian Capital Territory
AKI: Acute Kidney Injury
CCI: Charlson Comorbidity Index
ED: Emergency Department
GP: General Practitioner
ICD: International Classification of Diseases
LOS: Length of Stay
NSW: New South Wales
UR: Unit Record
US: United States
